# Comparative Prevalence of Immune Evasion Complex Genes Associated with β-Hemolysin Converting Bacteriophages in MRSA ST5 Isolates from Swine, Swine Facilities, Humans with Swine Contact, and Humans with No Swine Contact

**DOI:** 10.1371/journal.pone.0142832

**Published:** 2015-11-10

**Authors:** Samantha J. Hau, Jisun Sun, Peter R. Davies, Timothy S. Frana, Tracy L. Nicholson

**Affiliations:** 1 Department of Veterinary Diagnostic and Production Animal Medicine, College of Veterinary Medicine, Iowa State University, Ames, Iowa, United States of America; 2 Department of Veterinary Population Medicine, College of Veterinary Medicine, University of Minnesota, St Paul, Minnesota, United States of America; 3 National Animal Disease Center, Agricultural Research Service, United States Department of Agriculture, Ames, Iowa, United States of America; University Medical Center Utrecht, NETHERLANDS

## Abstract

Livestock associated methicillin-resistant *Staphylococcus aureus* (LA-MRSA) draws concern from the public health community because in some countries these organisms may represent the largest reservoir of MRSA outside hospital settings. Recent studies indicate LA-MRSA strains from swine are more genetically diverse than the first reported sequence type ST398. In the US, a diverse population of LA-MRSA is found including organisms of the ST398, ST9, and ST5 lineages. Occurrence of ST5 MRSA in swine is of particular concern since ST5 is among the most prevalent lineages causing clinical infections in humans. The prominence of ST5 in clinical disease is believed to result from acquisition of bacteriophages containing virulence or host-adapted genes including the immune-evasion cluster (IEC) genes carried by β-hemolysin converting bacteriophages, whose absence in LA-MRSA ST398 is thought to contribute to reduced rates of human infection and transmission associated with this lineage. The goal of this study was to investigate the prevalence of IEC genes associated with β-hemolysin converting bacteriophages in MRSA ST5 isolates obtained from agricultural sources, including swine, swine facilities, and humans with short- or long-term swine exposure. To gain a broader perspective, the prevalence of these genes in LA-MRSA ST5 strains was compared to the prevalence in clinical MRSA ST5 strains from humans with no known exposure to swine. IEC genes were not present in any of the tested MRSA ST5 strains from agricultural sources and the β-hemolysin gene was intact in these strains, indicating the bacteriophage’s absence. In contrast, the prevalence of the β-hemolysin converting bacteriophage in MRSA ST5 strains from humans with no exposure to swine was 90.4%. The absence of β-hemolysin converting bacteriophage in LA-MRSA ST5 isolates is consistent with previous reports evaluating ST398 strains and provides genetic evidence indicating LA-MRSA ST5 isolates may harbor a reduced capacity to cause severe disease in immunocompetent humans.

## Introduction

*Staphylococcus aureus* is a gram positive coccus that forms part of the normal nasal microflora in humans and other animal species. In developed countries, approximately one-quarter to one-third of healthy people harbor *S*. *aureus* in the nose, but prevalence appears to be lower in developing countries [1]. Although considered to be a commensal in the nasopharynx, *S*. *aureus* is an opportunistic pathogen causing a wide range of disease in humans. Skin and soft tissue infections are most commonly reported [2], but *S*. *aureus* also causes severe, invasive diseases including necrotizing pneumonia, bacteremia, osteomyelitis, and toxin mediated diseases such as toxic shock syndrome and staphylococcal enterotoxicosis. In 2005, the incidence rate for invasive MRSA infections per 100,000 individuals in the US was estimated to be 31.8 infections and 6.3 fatalities [3].

Resistance to methicillin was first reported in *S*. *aureus* in 1961 [4]. It is mediated by a mobile genetic element containing the *mecA* gene that confers resistance to methicillin and other β-lactam antibiotics. These isolates are designated methicillin resistant *S*. *aureus* (MRSA) and are difficult to treat, particularly if they have acquired multiple antibiotic resistance elements. Prevalence reports indicate MRSA may colonize as much as 1.5% of the healthy US population [5], and caused over 400,000 infections and millions of dollars in healthcare costs and lost productivity in 2009 [6].

Based on epidemiological characteristics, MRSA isolates are classified into three types: hospital-acquired (HA-MRSA), community-acquired (CA-MRSA), and livestock-associated (LA-MRSA). HA-MRSA isolates are obtained through contact in a healthcare setting. CA-MRSA isolates are not associated with a healthcare environment and are more commonly found in younger and healthy persons [2]. They are typically obtained from close contact environments, such as dormitories or athletic centers [7,8]. CA-MRSA isolates tend to possess fewer antimicrobial resistance elements, but exhibit increased virulence compared to HA-MRSA. The association between MRSA and swine was first reported in 2005 [9] and these isolates were referred to as LA-MRSA.

MRSA isolates are typically characterized by their genetic lineage through multi-locus sequence typing (MLST). MLST involves sequencing seven housekeeping genes (*arcC*, *aroE*, *glpF*, *gmk*, *pta*, *tpi*, *yqiL*) to obtain an allelic profile that defines the isolate’s sequence type (ST) [10]. STs present in the human population vary regionally and this trend is also true for animal populations, including swine. Initial reports of LA-MRSA from swine and other livestock species described isolates which all belonged to a novel MLST type (ST398). Subsequent research revealed a more complex epidemiology of *S*. *aureus* in pigs. The predominant swine-associated LA-MRSA lineage in Europe is ST398 [11], while in Asia ST9 isolates are most prevalent [12]. However, LA-MRSA isolates in the United States are a diverse population containing ST398, ST9, and ST5 isolates [13]. The presence of ST5 isolates in swine has raised additional public health concern because, in contrast to ST398 and ST9, ST5 is a highly successful and globally disseminated MRSA lineage in humans with both HA- and CA-MRSA clones reaching pandemic levels [14].

The clinical significance of ST5 strains is thought to be due to their ability to acquire mobile genetic elements (MGE) containing virulence factors or antibiotic resistance genes. Of particular importance is the β-hemolysin converting bacteriophage that is commonly acquired by MRSA ST5 isolates [14]. The β-hemolysin converting bacteriophage is a lysogenic phage that integrates into and disrupts the β-hemolysin gene of *S*. *aureus*. This bacteriophage contains an immune evasion cluster (IEC) encoding a combination of 1–4 known virulence factors that enhance the capacity of *S*. *aureus* strains to colonize, disseminate, and persist within a human host. These genes include: staphylococcal complement inhibitor (*scn*), chemotaxis inhibitory protein (*chp*), staphylokinase (*sak*), staphylococcal enterotoxin A (*sea*), and staphylococcal enterotoxin P (*sep*) [15]. These genes have been shown by *in vitro* assays to disrupt the normal function of the human immune system by inhibiting chemotaxis of phagocytes to the site of infection or inflammation (*chp*), preventing receptor-mediated phagocytosis (*sak*), inactivating antimicrobial peptides (*sak*), and inhibiting the complement cascade (*scn*) [16–18]. These proteins allow *S*. *aureus* to survive and replicate within host tissues causing local disease. Staphylokinase also acts through plasminogen activation to break down the extracellular matrix enabling bacterial dissemination from the initial site of infection [19]. Each of these proteins has been shown to be highly specific for human immune cells and serum proteins and are therefore considered human-specific virulence factors [16,17,20]. The toxin genes (*sea* and *sep*) are less specific to humans; however, they are important virulence determinants in disease. The enterotoxin proteins are superantigens, which when introduced systemically, non-specifically activate large populations of T cells in the host and cause dysregulation of the host’s adaptive immune response [21]. This limits the host’s ability to form an adaptive response specific to *S*. *aureus* antigens, preventing bacterial elimination [22]. While not all IEC genes are present within an individual prophage, the presence of a combination of several IEC genes confers increased virulence to an individual isolate. Previous studies indicate that prophage integration was present in 90.5% of human clinical isolates [15]; however, because not all genes are present on a given bacteriophage, the prevalence of individual genes differs.

In spite of the pathogenicity attributed to HA- and CA-MRSA ST5 isolates and detection of LA-MRSA ST5 in the nasal cavity of persons with swine contact, there are currently no known reports of clinical infection with ST5 isolates being attributed to animal contact. Epidemiological data has indicated that LA-MRSA ST398 isolates have reduced person-to-person transmission rates and are less virulent than their HA- and CA-MRSA counterparts [23,24]. Studies from pig dense regions in Europe suggest low risks of clinical infection with ST398 MRSA and there are few reports of severe clinical infections with LA-MRSA in people with animal contact despite high levels of exposure [25,26]. Previous studies focusing on ST398 isolates have demonstrated the absence of MGE associated virulence factors, including the IEC genes described here which is believed to contribute to the decreased pathogenicity and zoonotic potential of LA-MRSA isolates [27–29]. Reports showing a comparatively low prevalence of β-hemolysin converting bacteriophages in swine-associated ST398 isolates along with the lack of reports implicating swine exposure in MRSA ST5 related disease have led to the hypothesis that swine-associated LA-MRSA ST5 isolates would similarly have a low prevalence of prophage integration compared to their counterparts causing human clinical infections.

## Methods

### Strain acquisition

Swine associated isolates were obtained from Iowa State University [13] and the University of Minnesota. Sources for these isolates were swine (38 isolates), the environment within swine facilities (26 isolates), humans with short-term contact with swine (9 isolates), and swine veterinarians representing humans with long-term contact with swine (9 isolates). Clinical isolates from humans with no swine contact, representing both HA- and CA-MRSA, were obtained from the University of California Irvine (64 isolates) [30] and the University of California San Francisco (9 isolates). All isolates were MLST and Staphylococcal protein A (spa) typed prior to acquisition. Spa types can be found in S1 Table. ATCC strains Mu3 (ATCC #700698), Mu50 (ATCC #700699), Newman (ATCC #25904) were obtained for use as controls for the IEC genes, and a ST398 isolate from Iowa State University was used as a control for a strain encoding an intact β-hemolysin gene.

### DNA isolation

Strains were grown overnight on Trypticase Soy Agar (BD Biosciences, Sparks, MD) at 37°C to obtain isolated colonies. Individual colonies were selected to start an overnight culture of Trypticase Soy Broth (BD Biosciences, Sparks, MD). After 12–18 hours of growth, 750 μl of the liquid culture was pelleted and the supernatant was removed. The pelleted cells were stored at -80°C until DNA extraction. To isolate DNA, each pellet was resuspended in 200 μl 1x Phosphate Buffered Saline with 0.2 M EDTA. To lyse the cells, the following were added to each suspension: 12 μl of Lysozyme solution (Sigma, St. Louis, MO), 1 μl RNase (Roche, Mannheim, Germany), 7.5 μl Lysostaphin solution (Sigma, St. Louis, MO), and 7.5 μl Mutanolysin solution (Sigma, St. Louis, MO). The cells were then incubated for 1 hour at 37°C. Forty microliters of Proteinase K (Roche, Mannheim, Germany) was added and the suspension was incubated overnight at 55°C. The following day, a Roche High Pure PCR Template Preparation Kit (Roche, Mannheim, Germany) was used to isolate DNA according to the manufacturer’s protocol. The Elution Buffer containing DNA samples was centrifuged for 5 minutes at 8000xg to remove visible debris. The supernatant was transferred into a clean 1.5 mL tube and stored at 4°C until further analysis.

### PCR reactions

The primers, reaction conditions, and expected product size are listed in Table 1 [15,31–33]. Primers were designed based on a multiple sequence alignment of Mu3, Mu50, and Newman. PCR screening was conducted in 50 uL reaction volume using either AmpliTaq (Applied Biosystems, Carlsbad, CA) or AccuPrime Taq (Invitrogen, Carlsbad, CA) depending on the primer set. An MJ Research PCT-200 DNA Engine thermocycler (GMI, Ramsey, MN) was used for amplification using the following settings: 30 cycles of 30 second denaturation at 94°C, 30 seconds annealing at the temperature listed, 1 minute extension at 72°C (AmpliTaq) or 68°C (AccuPrime). PCR products were run on a 1% agarose gel, stained with ethidium bromide, and visualized using UV light. Nucleotide sequence determination of PCR products was completed by Sanger sequencing methods.

**Table 1:** Primer sets with reaction components, expected product size and primer source.

Primer	Sequence	Annealing Temp	DNA Polymerase Used	Expected Product Size	Primer Source
Int-F	GCTTTGAAATCAGCCTGTAGAGTC	54°	AmpliTaq	499 bp	This study
Hlb-R3	GTTGATGAGTAGCTACCTTCAGT	54°	AmpliTaq	499 bp	Jarraud et al [30]
Scn-F4	TGAGGCACAAGCTAGCACAAGCT	63°	AccuPrime	224 bp	This study
Scn-R4	TGAAGTTGATATTTTGCTTCTGACATTTTC	63°	AccuPrime	224 bp	This study
Sak-F2	TGAGGTAAGTGCATCAAGTTCA	53°	AmpliTaq	403 bp	Sung et al [31]
Sak-R2	CCTTTGTAATTAAGTTGAATCCAGG	53°	AmpliTaq	403 bp	Sung et al [31]
Chp-F	TTTACTTTTGAACCGTTTCCTAC	51.5°	AccuPrime	404 bp	Van Wamel et al [15]
Chp-R2	TGCATATTCATTAGTTTTTCCAGG	51.5°	AccuPrime	404 bp	Sung et al [31]
Sea-F5	GGTTATCAATGTGCGGGTGG	54°	AmpliTaq	322 bp	This study
Sea-R4	CAAATAAATCGTAATTAACCGAAGGTTC	54°	AmpliTaq	322 bp	Jarraud et al [30]
Sep-F2	GACCTTGGTTCAAAAGACACC	54°	AmpliTaq	275 bp	Diep et al [32]
Sep-R2	TGTCTTGACTGAAGGTCTAGC	54°	AmpliTaq	275 bp	Diep et al [32]
Hlb-TNF1	TATGTTATCGACCGTGTTGTATCC	58°	AmpliTaq	766 bp	This study
Hlb-TNR1	ATCCCATGGCTTAGGTTTTTCAGT	58°	AmpliTaq	766 bp	This study
Int-F	GCTTTGAAATCAGCCTGTAGAGTC	54°	AmpliTaq	499 bp	This study
Hlb-R3	GTTGATGAGTAGCTACCTTCAGT	54°	AmpliTaq	499 bp	Jarraud et al [30]
Scn-F4	TGAGGCACAAGCTAGCACAAGCT	63°	AccuPrime	224 bp	This study
Scn-R4	TGAAGTTGATATTTTGCTTCTGACATTTTC	63°	AccuPrime	224 bp	This study
Sak-F2	TGAGGTAAGTGCATCAAGTTCA	53°	AmpliTaq	403 bp	Sung et al [31]
Sak-R2	CCTTTGTAATTAAGTTGAATCCAGG	53°	AmpliTaq	403 bp	Sung et al [31]
Chp-F	TTTACTTTTGAACCGTTTCCTAC	51.5°	AccuPrime	404 bp	Van Wamel et al [15]
Chp-R2	TGCATATTCATTAGTTTTTCCAGG	51.5°	AccuPrime	404 bp	Sung et al [31]
Sea-F5	GGTTATCAATGTGCGGGTGG	54°	AmpliTaq	322 bp	This study
Sea-R4	CAAATAAATCGTAATTAACCGAAGGTTC	54°	AmpliTaq	322 bp	Jarraud et al [30]
Sep-F2	GACCTTGGTTCAAAAGACACC	54°	AmpliTaq	275 bp	Diep et al [32]
Sep-R2	TGTCTTGACTGAAGGTCTAGC	54°	AmpliTaq	275 bp	Diep et al [32]
Hlb-TNF1	TATGTTATCGACCGTGTTGTATCC	58°	AmpliTaq	766 bp	This study
Hlb-TNR1	ATCCCATGGCTTAGGTTTTTCAGT	58°	AmpliTaq	766 bp	This study

### Southern blotting

Genomic DNA (500ng) was digested overnight with BamH1 in a 25 uL reaction volume and run on a 1% agarose gel. Each gel was depurinated in 0.2M HCl for 10 minutes and rinsed with distilled water. It was then placed in denaturing solution for 1 hour followed by neutralizing solution for 1 hour. Each gel was set up for transfer via capillary action to a nylon membrane overnight. The DNA was crosslinked in UV light to the membrane. Prehybridization was done at 42°C for 2 hours and hybridized at 42°C overnight using a DIG labeled probe (Roche, Mannheim, Germany). The membrane was washed with 2X and 0.5X wash solution and blocked for 1 hour with maelic acid solution containing 5% powdered milk. It was then probed with anti-DIG antibody (Roche, Mannheim, Germany) at 1:10,000 in 5% powdered milk in maelic acid solution. The membrane was washed in washing buffer to remove excess antibody and CSPD (Roche, Mannheim, Germany) was added for visualization. Imaging was done using a myECLImager (Life Technologies, Grand Island, NY) or x-ray film development.

### Phage typing

Phage types were designated based on the complement of IEC genes present in the isolate as determined by PCR results. The typing scheme employed is the same as previously reported by van Wamel and colleagues [15]. Briefly, type A included isolates carrying the genes *sea*, *sak*, *chp*, and *scn*. Isolates containing *sak*, *chp*, and *scn* were designated type B. Type C comprised isolates containing *chp* and *scn*. Those isolates carrying *sea*, *sak*, and *scn* were labeled type D. Isolates harboring *sak* and *scn* were designated type E. Type F were isolates containing *sep*, *sak*, *chp*, and *scn*. Those isolates containing *sep*, *sak*, and *scn* were designated type G. Type H isolates were those containing only the *scn* gene as reported by Price and colleagues [27].

### Statistical analysis

The prevalence results were analyzed with a two-tailed Fisher’s exact test using the program GraphPad Prism (GraphPad Software, La Jolla, CA). A P value of 0.05 was used as the cutoff for statistical significance.

## Results

### IEC gene prevalence

PCR screens of the ST5 isolates were used to detect the presence of each of the five IEC genes known to be carried by β-hemolysin converting bacteriophages. Results of all screening tests are listed in Table 2 and S1 Table. The staphylococcal complement inhibitor (*scn*) gene was not found in any of the swine-associated ST5 isolates (0/82). It was detected in 90.4% (66/73) of the isolates from humans with no swine contact (p < 0.0001).

**Table 2:** Immune-evasion complex and β-hemolysin converting bacteriophage screening results for all isolates.

		Gene Tested						
Isolate Source		*scn*	*chp*	*sak*	*sea*	*sep*	*int*	Intact *hlb* ^a^
**Agricultural**	Human: short-term contact	0 (0/9)^b^	0 (0/9)	0 (0/9)	0 (0/9)	0 (0/9)	0 (0/9)	100 (9/9)
**Agricultural**	Human: long-term contact	0 (0/9)	0 (0/9)	0 (0/9)	0 (0/9)	0 (0/9)	0 (0/9)	100 (9/9)
**Agricultural**	Pig	0 (0/38)	0 (0/38)	0 (0/38)	0 (0/38)	0 (0/38)	0 (0/38)	100 (38/38)
**Agricultural**	Environment	0 (0/26)	0 (0/26)	0 (0/26)	0 (0/26)	0 (0/26)	0 (0/26)	100 (26/26)
**Clinical**		90.4 (66/73)	87.7 (64/73)	90.4 (66/73)	1.4 (1/73)	37.0 (27/73)	90.4 (66/73)	9.6 (7/73)

^a^ Data reported represent the results from Southern blotting.

^b^ Data reported as percent of isolates positive for each gene tested. Number of positive isolates is noted in parenthesis.

The gene for the chemotaxis inhibitory protein (*chp*) was absent in the swine-associated isolates (0/82), but was detected in 87.7% (64/73) of isolates from humans with no swine contact (p < 0.0001).

The staphylokinase (*sak*) gene was also lacking in all of the swine-associated isolates (0/82), but found to be present in 90.4% (66/73) of isolates from humans with no swine contact (p < 0.0001).

The gene encoding staphylococcal enterotoxin-like P (*sep*) was absent from all swine-associated isolates (0/82), but detected in 37.0% (27/73) of isolates from humans with no swine contact (p < 0.0001).

The staphylococcal enterotoxin A (*sea*) gene was not found in any of the swine-associated isolates (0/82), and the prevalence of this gene was also low (1.4%, 1/73) in isolates from humans with no swine contact (p = 0.4744).

### β-hemolysin converting bacteriophage prevalence via integrase gene screening

**Fig 1:**
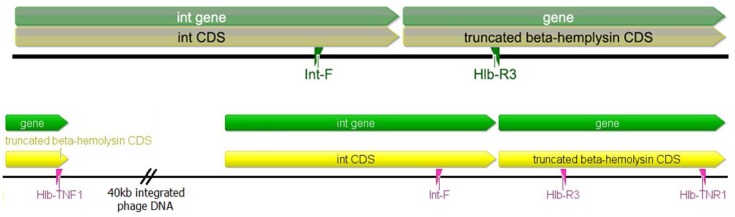
Location of primers used for PCR used to test for the presence of the integrase gene and intact β-hemolysin gene. The integrase gene associated with the β-hemolysin converting bacteriophage (int CDS) and the 5’ end of the disrupted β-hemolysin gene (truncated beta-hemolysin CDS) are shown along with the primer specific for the the integrase gene (Int-F) and the primer specific for the β-hemolysin gene (Hlb-R3). The primers used for detection of an intact β-hemolysin gene (Hlb-TNF1 and Hlb-TNR1) spanned the disrupted portion of the gene and no product should be generated when the phage has integrated.

Although PCR results for the IEC genes indicated the presence or absence of the β-hemolysin converting bacteriophage in the tested isolates, PCR analysis for the integrase gene insertion site was used to verify the presence of an integrated phage. The integrase gene enables lysogenic bacteriophages to integrate into the bacterial genome. Due to the high degree of nucleotide sequence conservation of bacteriophage integrase genes, the primers used for this PCR analysis were developed to create a product that spanned the 3’ end of the integrase gene and the 5’ end of the β-hemolysin gene (Fig 1). This ensured specificity to the integrase gene of the β-hemolysin converting bacteriophage. None of the swine isolates were found to contain the integrase/β-hemolysin gene junction (0/82), while the gene was present in 90.4% (66/73) of the isolates from humans with no swine contact (p < 0.0001).

### β-hemolysin gene analysis

**Fig 2:**
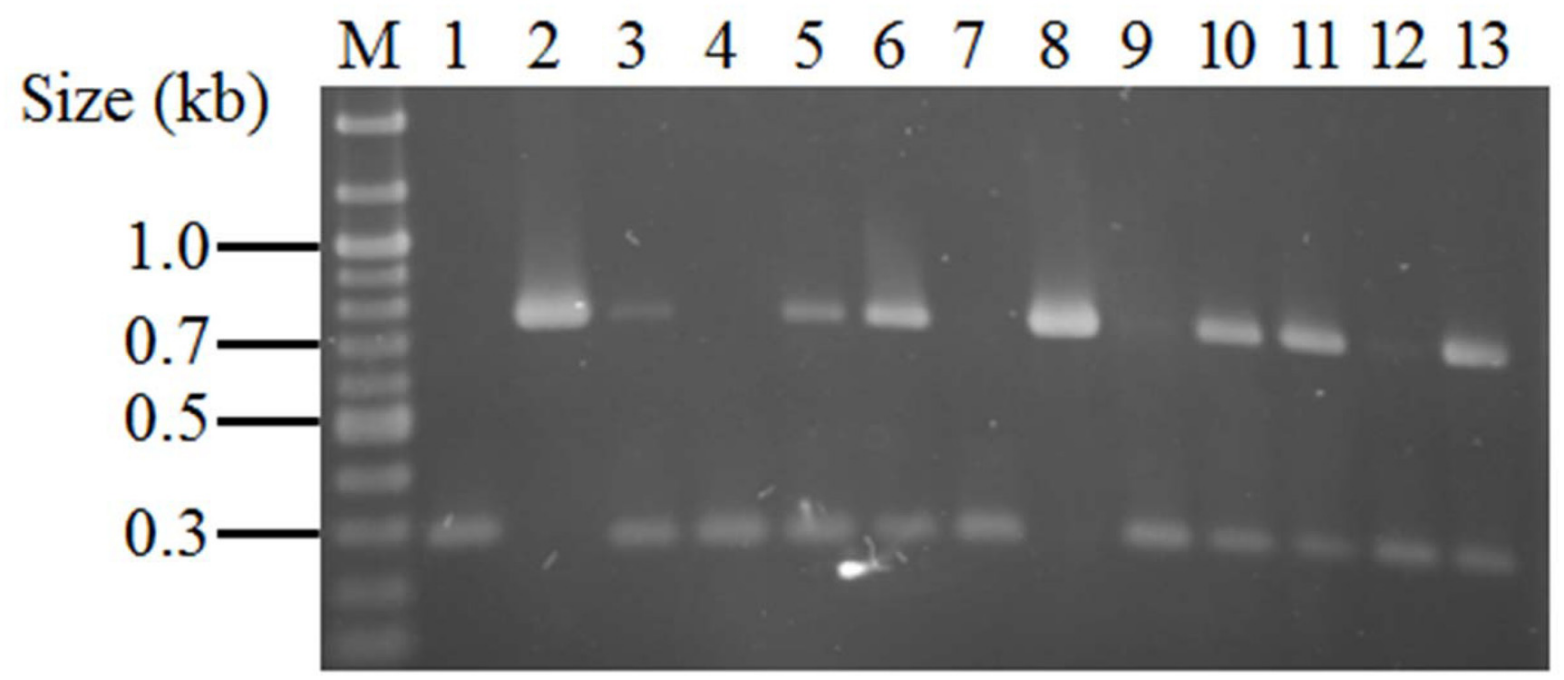
Agarose gel electrophoresis demonstrating inconclusive banding pattern resulting from PCR used to test an intact β-hemolysin gene in isolates obtained from humans with no swine contact. Those isolates producing only a 750bp band were found to contain an intact β-hemolysin gene, while isolates producing only a 300bp band or both bands were found to contain a disrupted β-hemolysin gene. A disrupted β-hemolysin gene is represented by the negative control ST8 Newman (lane 1), which produced a band 300bp in size. An intact β-hemolysin gene is represented by the positive control ST398 (lane 2), which produced a band 750bp in size. Of the isolates from humans with no swine contact, 9.6% (7/73) produced a 750bp band (lane 8) and 32.9% (24/73) produced a 300bp band (lane 4, 7). However, 57.5% (42/73) of the isolates produced both a 750bp and a 300bp band (lane 3, 5, 6, 9–13).

To further confirm the absence of the β-hemolysin converting bacteriophage in the isolates tested, PCR screening was undertaken using primers Hlb-TNF1 and Hlb-TNR1 (Fig 1). An ST398 strain was used as a control for the absence of the bacteriophage and produced a 750 base pair product, while the controls for the presence of the bacteriophage, ST8 Newman, ST5 Mu3, ST5 Mu50, produced a 300 base pair product. After further analysis, the nucleotide sequence of 300 base pair product produced by the control isolates containing the bacteriophage was determined to be a portion of bacteriophage DNA at the 5’ end of the integrated sequence. All swine-associated isolates produced a band 750 base pairs in length (82/82). These results demonstrate that the β-hemolysin gene is intact and no bacteriophage is present in these strains. Of isolates from humans with no swine contact, 9.6% (7/73) produced a band 750 base pairs in length. However, only 32.9% (24/73) of the isolates produced a single band 300 base pairs in length. Many of the isolates (57.5%, 42/73) produced two bands, one at 750 base pairs and the other at 300 base pairs in length (Fig 2). After comparing these results with the PCR results for the IEC genes and the integrase gene, it was determined that the isolates producing a 300 base pair band, even with the presence of a 750 base pair band, were carrying a disrupted β-hemolysin gene. One explanation for the multiple PCR products is that they may have been produced due to the induction of beta-converting phage during bacterial culture, subsequently leading to the loss of phage in a sub-population of bacterial cells.

**Fig 3:**
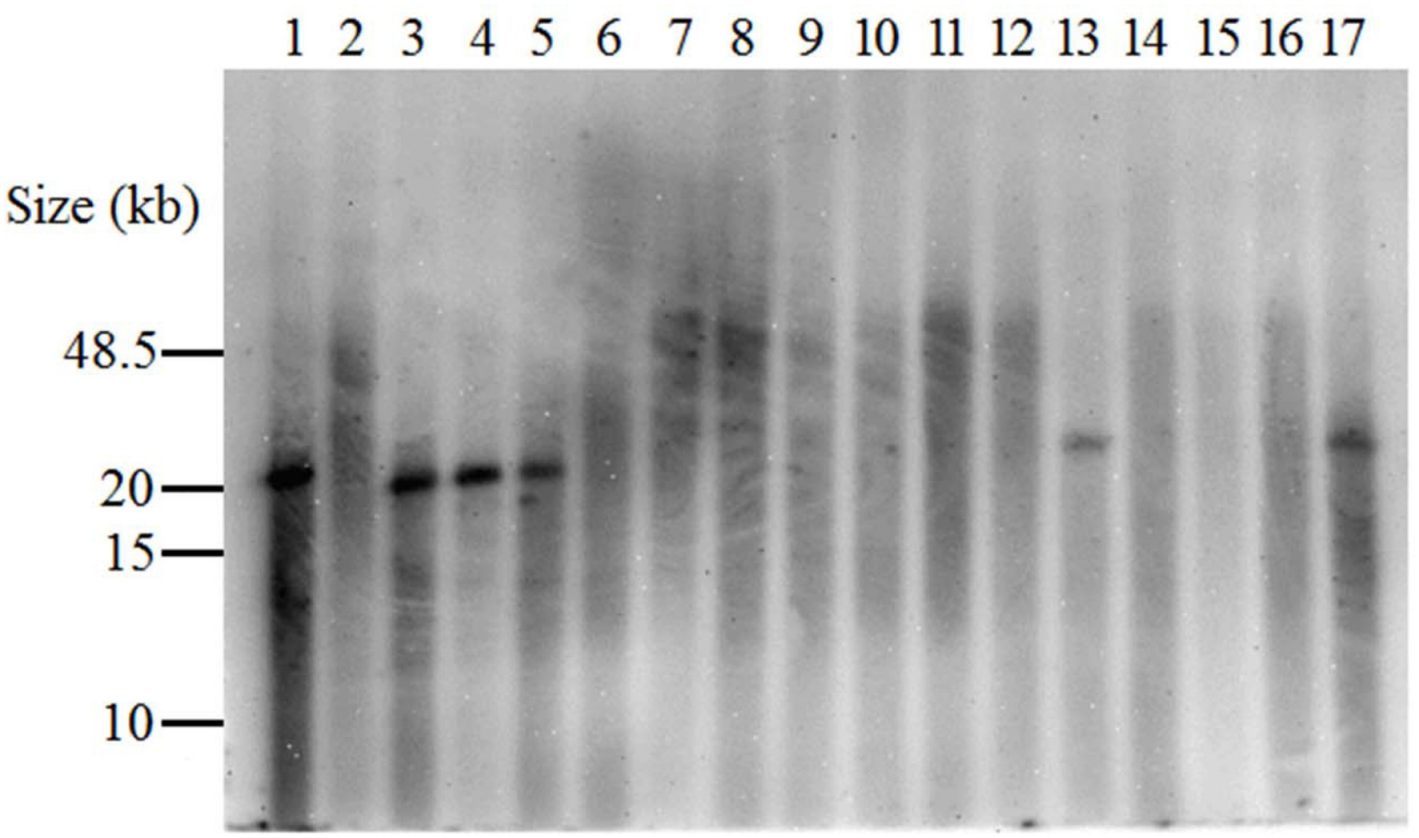
Southern blot demonstrating the presence of an intact or disrupted β-hemolysin gene. Isolates containing an intact β-hemolysin gene produced a distinct band (20kb) as seen with the control isolate ST398 that lacked the bacteriophage (lane 1). Isolates containing a disrupted β-hemolysin gene did not produce a distinct band at 20kb and background extended in the lanes to a size greater than 40kb, as seen with the control ST8 Newman (lane 2). Swine-associated MRSA ST5 isolates are represented by lanes 3–5 and MRSA ST5 from humans with no swine contact are represented by lanes 6–17. Lanes 13 and 17 contained isolates from humans with no swine contact bearing an intact β-hemolysin gene.

Due to the inconclusive banding pattern, Southern blotting was used to confirm whether the β-hemolysin gene was intact or disrupted using a probe derived from the 750bp PCR product of ST398 and the Hlb-TNF1 and Hlb-TNR1 primers. The restriction enzyme BamH1 was selected to digest the genomic DNA because no restriction sites were present within the β-hemolysin gene or any of the bacteriophage genes (confirmed using Newman, Mu3, and Mu50 genomes in GenBank accession numbers NC_009641, NC_009782, NC_002758 respectively). The genome fragments produced by BamH1 digestion of isolates with an intact and disrupted β-hemolysin genes were approximately 20kb and 65kb respectively and were readily distinguished during analysis of the Southern blots (Fig 3). Intact genes produced a distinct band around 20kb, while disrupted genes showed background extending beyond 48kb with no distinct band. The lack of a distinct band seen with the disrupted genes was attributed to reduced annealing strength of the probe to the disrupted β-hemolysin gene and the reduced transfer rate seen with larger band sizes. Southern blotting was able to confirm β-hemolysin gene disruption in 90.4% (66/73) of the isolates from humans with no swine contact. The β-hemolysin gene was intact in 9.6% (7/73) of isolates from humans with no swine contact and in 100% (82/82) of the swine-associated isolates (p < 0.0001).

### Phage typing

The different combinations of the immune evasion complex genes carried by the β-hemolysin converting bacteriophage have been previously defined into seven types (A-G) by van Wamel and colleagues [15]. A novel phage type was discovered by Price and colleagues and will be referred to here as type H [27]. The phage types found in both studies can be found in Table 3 along with the prevalence rate found in the isolates from humans with no swine contact analyzed in this study. The ST5 isolates evaluated in this study were found to contain primarily type B (50%, 36/73) and type F (37.0%, 27/73) prophages.

**Table 3:** Phage types in human clinical isolates.

Phage Type	Genes	van Wamel et al [15]	Price et al [26]	This Study
A	sea—sak—chp—scn	^a^12.2 (11)	0 (0)	1.4 (1)
B	sak—chp—scn	26.7 (24)	31.6 (6)	49.3 (36)
C	chp—scn	13.3 (12)	52.6 (10)	0 (0)
D	sea—sak— —scn	15.6 (14)	0 (0)	0 (0)
E	sak— —scn	14.4 (13)	0 (0)	2.7 (2)
F	sep—sak—chp—scn	4.4 (4)	0 (0)	37.0 (27)
G	sep—sak— —scn	3.3 (3)	0 (0)	0 (0)
H	scn	0 (0)	10.5 (2)	0 (0)
None	None	10 (9)	5.3 (1)	9.6 (7)

^a^ Data are reported as the percent of isolates of each phage type out of the number of isolates evaluated. The number of isolates found of each phage type is listed in parenthesis.

## Discussion

It has long been known that *S*. *aureus* commonly colonizes many mammalian and avian species and particular lineages are more adapted to different host species [34]. The recent recognition that livestock may represent a substantial reservoir of MRSA and people having regular contact with animals were commonly colonized with LA-MRSA isolates represented a shift in MRSA epidemiology and raised urgent questions about the public health significance of these organisms. Fundamental questions remain about the ability of *S*. *aureus* lineages adapted to animals to both colonize and cause disease in humans. The capacity of ST398 LA-MRSA to cause clinical disease in humans is established, but reports of severe infections in people with occupational exposure to livestock remain uncommon, despite continued exposure to these organisms. There is increasing evidence that ST398 MRSA isolates of animal origin are less likely to be transmitted between people and are less likely to be associated with severe infections than are human adapted variants. Genomic studies have indicated that distinct livestock and human variants are identifiable even within a given sequence type and spa type, such as ST398/t571 and ST1/t127 [35,36]. More specifically, the absence of MGE associated virulence factors, including IEC genes has been linked to host adaptation and loss of virulence in ST398 LA-MRSA [27–29].

The MRSA lineages ST398 and ST9, which predominate in swine populations in Europe and Asia respectively, do not appear to have a significant impact on human health in the US. Unlike in Europe and Asia where ST5 MRSA have only rarely been reported, several studies indicated that ST5 S. aureus (both MRSA and MSSA) are relatively common in the North American swine industry [13,37–40]. Because the ST5 lineage is a major contributor to both hospital and community associated MRSA and MSSA infections in this country and worldwide [14,41], it is important to address the question about the potential contribution, if any, of the swine reservoir to the burden of clinical disease associated with ST5 *S*. *aureus*.

At this time, no human disease due to swine-associated LA-MRSA ST5 isolates has been reported. This may be due to differences in the composition of the accessory genome seen in LA-MRSA versus HA- and CA-MRSA isolates, similar to that previously noted in ST398 isolates [27,29]. HA- and CA-MRSA ST5 isolates are known to carry several MGE that enhance their virulence and antibiotic resistance, which have contributed to their dissemination and pathogenicity [14].

This is the first report examining β-hemolysin converting bacteriophage prevalence within human clinical MRSA ST5 isolates specifically. The incidence of prophage integration in ST5 isolates obtained from humans with no swine contact was consistent with that found in previous reports of human clinical *S*. *aureus* isolates, both MRSA and methicillin-susceptible [15,27,29]. Additionally, this is the first study investigating the prevalence of the β-hemolysin converting bacteriophage in LA-MRSA ST5 isolates. The results obtained in this study are consistent with previous publications, and comparative statistical analysis showed no significant difference (p = 0.3987) in the prevalence of β-hemolysin converting bacteriophages between LA-MRSA ST5 isolates evaluated in this study (0/82) and LA-MRSA ST398 isolates evaluated previously (1/63) by Price and colleagues [27].

Due to the restricted host specificity of the most prevalent genes (*sak*, *scn*, *chp*), it has been suggested that this prophage may be absent in MRSA isolates after adapting to a livestock niche [27]. These genes would not confer an advantage during colonization or disease development in livestock species and are therefore unnecessary to retain within the genome of LA-MRSA isolates. The loss of these important virulence factors is likely one of the reasons LA-MRSA isolates are rarely known to cause invasive disease in immunocompetent humans. The absence of IEC genes carried by β-hemolysin converting bacteriophages in LA-MRSA ST398 and ST5 strains parallels the findings for poultry adapted ST5 strains in that the human-specific IEC genes were lost and subsequently replaced by genes encoding avian-specific factors after the human-to-poultry transition [42]. However, unlike poultry adapted ST5 strains, swine-associated LA-MRSA ST398 and ST5 strains harbor an intact β-hemolysin gene, indicating that the bacteriophage is absent from these strains rather than being replaced by genes encoding swine-specific factors.

There were several interesting differences noted between the prevalence of phage types found in the human clinical isolates evaluated in this study compared to those previously reported by van Wamel and colleagues (Table 3). Their initial investigation and description of the β-hemolysin converting bacteriophage types was completed using 85 clinical *S*. *aureus* isolates from several hospitals in the Netherlands and 5 *S*. *aureus* reference strains [15]. These isolates were not characterized using MLST typing and are therefore thought to have a more diverse genetic background than the isolates evaluated here. Specifically, the enterotoxin A (*sea*) and enterotoxin-like P (*sep*) gene prevalence varied considerably between these investigations. In this study, significantly more (p < 0.0001) isolates were found to harbor the *sep* gene and significantly less (p < 0.0001) isolates were found to harbor the *sea* gene compared to the prevalences previously reported. The results reported here correlate with a decrease in the prevalence of phage types A and D and an increase in phage type F. This was surprising in that *sea* is considered to be the most common enterotoxin involved in staphylococcal food borne illness [21]. The increased prevalence of *sep* seen in this study may indicate a larger role in the development of clinical disease than previously described. Additionally, significantly more isolates were found to harbor the *chp* gene (p < 0.0001) and the *sak* gene (p = 0.0226) in this study. These results correlate with an increase in prevalence of the type B and F prophages that contain the three innate immune evasion genes. Variability in phage types seen within this population of clinical MRSA ST5 isolates indicates multiple acquisitions of the β-hemolysin converting bacteriophage even within a regional population. The differences in phage prevalence reported in this study compared to the van Wamel study may be due to the sample populations evaluated. The isolates evaluated previously were clinical isolates from several hospitals in the Netherlands and no MLST types were indicated. In contrast, the isolates evaluated in this study comprised of 73 clinical ST5 isolates from University of California associated medical facilities. The differences in phage prevalence reported could be attributed to the disease profile of the isolates, the narrowed genetic background of the isolates evaluated, or a regional difference (California versus the Netherlands) not previously noted.

ST5 *S*. *aureus* appear to be widespread in the North American swine population and have likely been endemic in this reservoir for some time, yet livestock contact has not been identified as a risk factor for clinical staphylococcal disease [43]. This investigation identified clear genomic differences between ST5 MRSA isolates linked to swine and isolates from human clinical infections. These differences parallel previous observations with ST398 isolates. We hypothesize that the genetic changes observed may reflect general processes related to host adaptation of *S*. *aureus* to pigs. More extensive genomic investigations of ST5 *S*. *aureus* in pigs are warranted, as is investigation of other lineages *S*. *aureus* associated with pigs such as ST9.

## Supporting Information

S1 Table Individual Isolate Information. (XLSX)
